# FXR1 can bind with the CFIm25/CFIm68 complex and promote the progression of urothelial carcinoma of the bladder by stabilizing TRAF1 mRNA

**DOI:** 10.1038/s41419-022-04614-1

**Published:** 2022-02-22

**Authors:** Minhua Deng, Ning Wang, Zhiyong Li, Rixin Chen, Jinling Duan, Yulu Peng, Zeshen Wu, Zhiling Zhang, Lijuan Jiang, Xianchong Zheng, Dan Xie, Wensu Wei, Zhuowei Liu, Fangjian Zhou

**Affiliations:** 1 State Key Laboratory of Oncology in South China, Collaborative Innovation Center for Cancer Medicine, Sun Yat-Sen University Cancer Center, No. 651, Dongfeng Road East, Guangzhou, China; 2 Department of Urology, Sun Yat-Sen University Cancer Center, No. 651, Dongfeng Road East, Guangzhou, China; 3 Department of Thoracic Surgery, Guangdong Provincial People’s Hospital, Guangdong Academy of Medical Sciences, Guangzhou, China; 4 Department of Pathology, Sun Yat-Sen University Cancer Center, No. 651, Dongfeng Road East, Guangzhou, China

**Keywords:** Bladder cancer, Oncogenes

## Abstract

RNA-binding proteins (RBPs) are key regulators of gene expression. RBP dysregulation is reported to play essential roles in tumorigenesis. However, the role of RBPs in urothelial carcinoma of the bladder (UCB) is only starting to be unveiled. Here, we comprehensively assessed the mRNA expression landscape of 104 RBPs from two independent UCB cohorts, Sun Yat-sen University Cancer Center (SYSUCC) and The Cancer Genome Atlas (TCGA). Fragile X-related gene 1 (FXR1) was identified as a novel cancer driver gene in UCB. FXR1 overexpression was found to be related to the poor survival rate in the SYSUCC and TCGA cohorts. Functionally, FXR1 promotes UCB proliferation and tumorigenesis. Mechanistically, FXR1 serves as a platform to recruit CFIm25 and CFIm68, forming a novel 3′ processing machinery that functions in sequence-specific poly(A) site recognition. FXR1 affects the 3′ processing of Tumor necrosis factor receptor-associated factor 1 (TRAF1) mRNA, which leads to nuclear stabilization. The novel regulatory relationship between FXR1 and TRAF1 can enhance cell proliferation and suppress apoptosis. Our data collectively highlight the novel regulatory role of FXR1 in *TRAF1* 3′ processing as an important determinant of UCB oncogenesis. Our study provides new insight into RBP function and provides a potential therapeutic target for UCB.

## Introduction

Urothelial carcinomas are the most common cancers in urological systems, and more than 90% are urothelial carcinomas of the bladder (UCBs) [1]. According to research, in the United States, approximately 81,400 new bladder cancer cases and 17,980 new death cases were expected in 2020 [2]. About 30% of newly diagnosed UCB patients have muscle-invasive bladder cancer (MIBC), which includes localized and metastatic disease [3]. For advanced MIBC, even though patients receive adjuvant chemotherapy after standard radical cystectomy, the 5-year overall survival (OS) rate is still less than 40% [4]. For the purpose of improving the prognosis of UCB patients, exploring the deeper molecular mechanisms and finding effective anticancer treatment is urgent.

RNA-binding proteins (RBPs) exert critical and multifaceted roles in gene regulation processes, such as RNA splicing, cleavage and polyadenylation, transport, translation, and stability [5–9]. RBPs function by binding to their target RNA through specific RNA-binding domains (RBDs) and forming ribonucleoprotein (RNP) complexes. Notably, although RBPs interact with various classes of RNAs, such as ribosomal RNAs, snRNA, snoRNA, tRNAs, mRNAs, and non-coding RNAs, approximately half of RBPs exert their distinct roles by regulating mRNA fate [10]. Recent high-throughput screens have identified more than 1500 RBPs in the human genome [10]. However, only a small proportion of them have been functionally characterized.

Given the critical role of RBPs in many steps of gene expression, mutations or expression alterations of RBPs can frequently cause alterations in multiple biological and pathological processes that lead to diseases, including cancer [11–14]. RBP dysregulation can facilitate tumorigenesis by enhancing oncogene expression or inhibiting tumor suppressor expression. However, the function of RBPs in bladder cancer is still rarely elucidated.

In this research, we analyzed RNA-sequencing (RNA-seq) of 22 pairs of UCB and normal samples from Sun Yat-Sen University Cancer Center (SYSUCC) and 19 paired UCBs from Tumor Cancer Genome Atlas (TCGA). We reported a comprehensive mRNA expression profile of 104 RBPs from these two independent UCB cohorts. We identified that Fragile X-related gene 1 (FXR1), a potential cancer driver gene, is frequently upregulated in UCB and other cancer types. High expression of FXR1 was positively associated with worse OS in SYSUCC and TCGA cohorts. FXR1 is necessary for cancer cell viability and is sufficient to drive tumorigenesis. Mechanistic studies revealed that FXR1 serves as a platform to recruit CFIm25 and CFIm68, forming a novel complex for mRNA 3′ end processing that functions in sequence-specific poly(A) site recognition. FXR1 affects the 3′ processing of Tumor necrosis factor receptor-associated factor 1 (TRAF1) mRNA, which leads to nuclear stabilization. Importantly, FXR1 regulates the tumorigenic functions of TRAF1 in UCB cells, enhancing cell proliferation and suppressing apoptosis. Our findings reveal that consistently upregulated FXR1 expression is a new cause of UCB oncogenesis. Our study provides novel insight into RBP function and implies a potential therapeutic target for UCB.

## Results

### FXR1 is a consistently upregulated RBP in human cancer

**Fig. 1:**
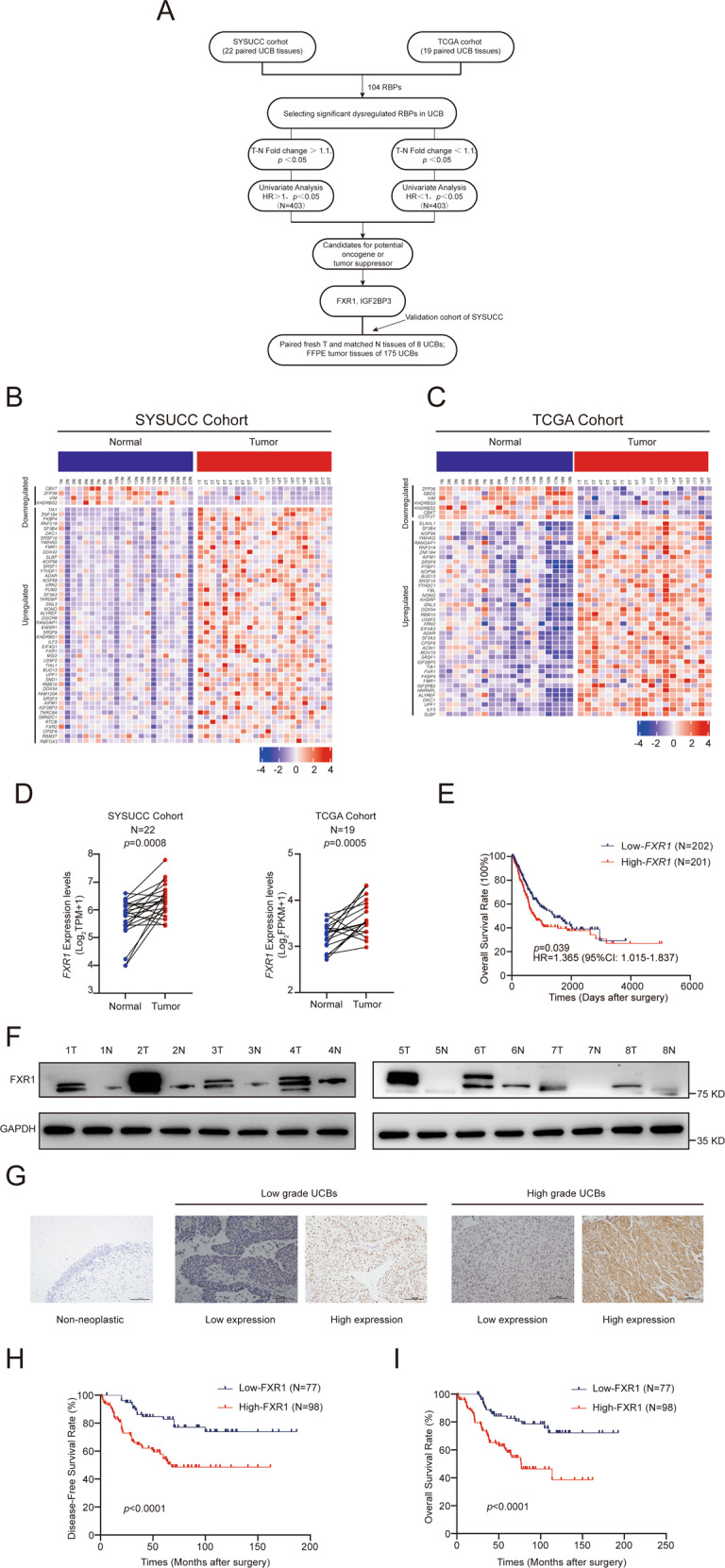
RBPs are frequently upregulated in UCB tissue and FXR1 upregulation predicts poor DFS and OS in UCB patients from SYSUCC and TCGA cohorts. **A** Flowchart illustrating the different steps in the analysis of significantly dysregulated RBPs in UCB patients. **B**, **C** Heatmap showing the mRNA expression levels of significantly dysregulated RBPs in the SYSUCC (**B**) and TCGA (**C**) cohorts. **D** Distribution of *FXR1* mRNA expression in tumors compared with matched normal samples from the SYSUCC (left) and TCGA (right) cohorts. **E** Survival analysis indicates the correlation between high *FXR1* expression levels and poor OS rates in TCGA UCB patients. HR hazard ratio, CI confidence interval. **F** Western blot showing the upregulated expression of FXR1 in eight paired UCB tissues. **G** Representative images of IHC staining for FXR1 in nonneoplastic bladder tissue, low-grade UCB, and high-grade UCB. **H**, **I** Survival analysis showing that high expression of FXR1 predicts poor DFS and OS rates in SYSUCC UCB patients.

To assess the dysregulation of RBPs in UCB, we analyzed the mRNA expression of 104 RBPs in 22 pairs of UCB and normal samples from SYSUCC and 19 paired UCB samples from TCGA (Fig. 1A). RNA-seq of the SYSUCC data identified 54 dysregulated RBPs by comparing the mRNA expression levels in tumors to those in adjacent normal tissues (paired *t*-test, *p* < 0.05, |fold change | >1.1). Interestingly, we found that more RBPs were upregulated in UCB tissues (Fig. 1B and Supplementary Fig 1A), including 50 upregulated RBPs and 4 downregulated RBPs. We further analyzed the expression of 104 RBPs in TCGA UCB cohorts. We found that 41 RBPs were upregulated and 7 RBPs were downregulated in UCB tumors from TCGA (paired *t*-test, *p* < 0.05, |fold change | >1.1, Fig. 1C and Supplementary Fig 1B). Among 32 consistently upregulated RBPs in both the SYSUCC and TCGA cohorts, *FXR1* and *IGF2BP3* displayed an inverse correlation with OS (high expression levels indicated worse OS) in the TCGA UCB cohort according to Cox hazard analysis (*p* < 0.05) (Fig. 1D, E and Supplementary Fig 1C, D). In addition, high *FXR1* expression strongly increased the risk of death from disease (hazard ratio = 1.365) (Fig. 1E). Together, these results suggest that the upregulation of *FXR1* can contribute to cancer development.

To further explore the correlation between FXR1 expression and clinical outcomes in UCB patients, we first examined the protein expression in eight pairs of UCB and adjacent nonneoplastic tissues. We found that FXR1 was frequently upregulated in UCB tumors (Fig. 1F). Immunohistochemistry (IHC) staining of 175 UCB tissues and 8 matched noncancerous bladder tissues obtained from SYSUCC revealed high expression of FXR1 in 98 of 175 samples (56%); whereas in noncancerous bladder tissues, FXR1 was expressed at low levels (Fig. 1G). High expression of FXR1 in UCB patients was positively associated with advanced T and N stage, tumor grade, and tumor volume (*p* < 0.05, Supplementary Table S1). Additionally, high FXR1 expression predicted poor disease-free survival (DFS) and OS in UCB patients (*p* < 0.05, Fig. 1H, I). The results of multivariate regression analysis revealed that FXR1 is an independent prognostic factor for UCB patients (Table 1). These results suggest that upregulation of FXR1 is a frequent oncogenic event in UCB pathogenesis.

**Table 1:** Univariate and multivariate analysis of different prognostic parameters in 175 patients with UCB in SYSUCC.

Variable	All cases	Univariate analysis^a^	*P* value	Multivariate analysis^b^	*P* value
		HR (95% CI)		HR (95% CI)	
*Age (years)*			0.248		
≤60	86	1			
>60	89	1.357 (0.808–2.279)			
*Gender*			0.501		
Female	21	1			
Male	154	1.370 (0.547–3.430)			
*Smoking history*			0.872		
No	85	1			
Yes	90	1.043 (0.622–1.750)			
*pT status*			0.168		0.433
T1	33	1		1	
T2–T4	142	1.691 (0.801–3.568)		0.725 (0.325–1.619)	
pN status			0.001		0.012
pN−	142	1		1	
pN+	33	3.737 (2.058–6.785)		2.315 (1.203–4.454)	
*Histological grade (WHO, 2004)*			0.013		0.098
Low grade	59	1		1	
High grade	116	2.197 (1.184–4.077)		1.786 (0.899–3.548)	
*FXR1*			0.001		0.004
Low expression	77	1		1	
Overexpression	98	3.084 (1.721–5.527)		2.471 (1.336–4.569)	
*Tumor volume*			0.014		0.019
≤3 cm	82	1		1	
>3 cm	93	1.970 (1.146–3.387)		1.980 (1.121–3.497)	
*Recurrence*			0.001		0.001
No	113	1		1	
Yes	62	6.060 (3.362–10.925)		6.504 (3.572–11.842)	

^a^Univariate Cox regression model.

^b^Multivariate Cox regression model.

*CI* confidence interval, *HR* hazards ratio, *UCB* urothelial carcinoma of the bladder.

To determine whether upregulation of FXR1 frequently occurs in other solid tumors, we analyzed the mRNA expression of *FXR1* from TCGA across 28 cancer types by paired *t*-test. Due to an insufficient number of paired normal tissues, 14 cancer types were excluded (*n* < 6). We found that FXR1 was predominantly upregulated in 10 out of 14 cancer types, including lung squamous cell carcinoma, lung adenocarcinoma, kidney clear cell carcinoma, stomach cancer, rectal cancer, liver cancer, head and neck cancer, colon cancer, bile duct cancer, and esophageal cancer (Supplementary Fig 1E). Together, these results revealed that upregulation of FXR1 could function in tumorigenesis in many types of human solid tumors, including UCB.

### FXR1 increased UCB cell proliferation capacity in vitro and in vivo

**Fig. 2:**
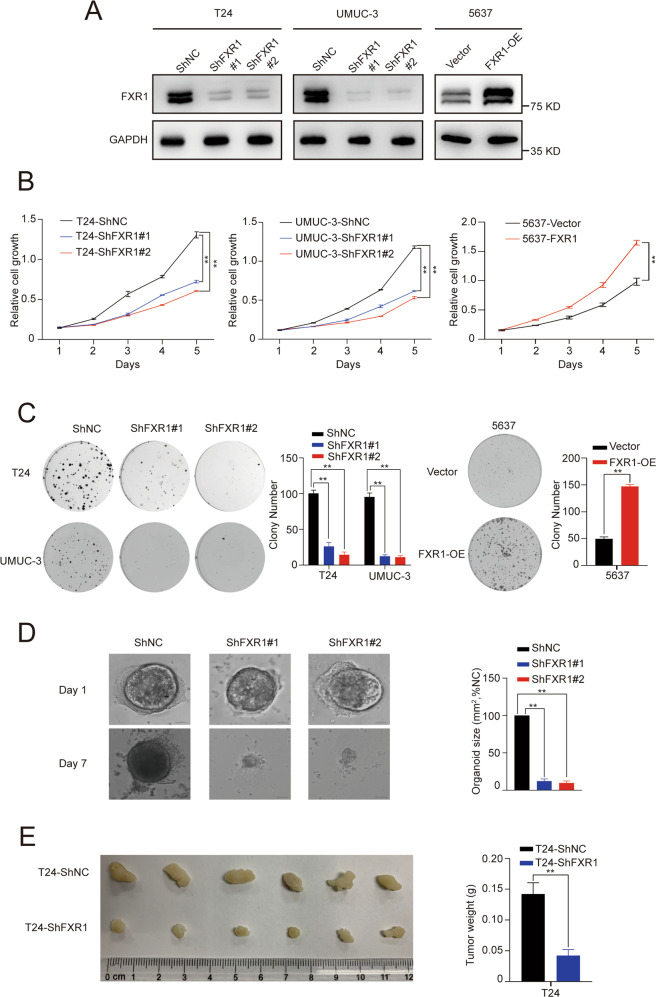
FXR1 promotes UCB cell proliferation in vitro and in vivo. **A** Western blot showing the efficiency of FXR1 knockdown or overexpression in the indicated UCB cell lines. **B** Growth rate of UCB cells in control or FXR1 knockdown (left) or FXR1 overexpression cells (right) as revealed. Data are presented as the mean ± SD, *n* = 3, **p < 0.01 (Student’s *t*-test). **C** Representative images of colony foci formation in monolayer culture of control or FXR1 knockdown (left) or FXR1 overexpression cells (right). The results for quantitative analysis of foci numbers are shown in the adjacent graphs. Data are presented as the mean ± SD, *n* = 3, **p < 0.01 (Student’s *t*-test). **D** Representative images of organoid growth after infection with control or FXR1 shRNA lentivirus. Quantitative analysis of organoid size is shown in the adjacent graphs. Data are presented as the mean ± SD, *n* = 3, **p < 0.01 (Student’s *t*-test). **E** Image of the xenograft tumors formed in Balb/c nude mice injected with T24-shFXR1 or T24-shNC cells. The weights of xenograft tumors are shown in the right panel. Data are shown as mean ± SD, *n* = 6, **p < 0.01 (independent Student’s *t*-test).

To explore the biological functions of FXR1 in UCB cells, we stably knocked down FXR1 in T24 and UMUC-3 cell lines, which had high baseline expression of FXR1 (Fig. 2A, Supplementary Fig 2A). We found that knockdown of FXR1 decreased the cell proliferation capacity, as determined by CCK-8 and colony formation assays (Fig. 2B, C). However, stable overexpression of FXR1 in 5637 cells, which had low baseline expression of FXR1, markedly increased cell growth and colony formation ability (Fig. 2B, C).

To further characterize the pathological role of FXR1, we generated a patient-derived organoid line of bladder cancer in three-dimensional culture to recapitulate the pathological and genomic features of human bladder cancer. The results showed that FXR1 knockdown via short hairpin RNA (shRNA) lentiviral infection resulted in markedly decreased tumor organoid growth (Fig. 2D). Supporting this, the results from a subcutaneous xenograft mouse model revealed that FXR1 knockdown dramatically reduced the size and weight of subcutaneous xenograft tumors (Fig. 2E).

### Knockdown of FXR1 promoted the apoptosis of UCB cells

**Fig. 3:**
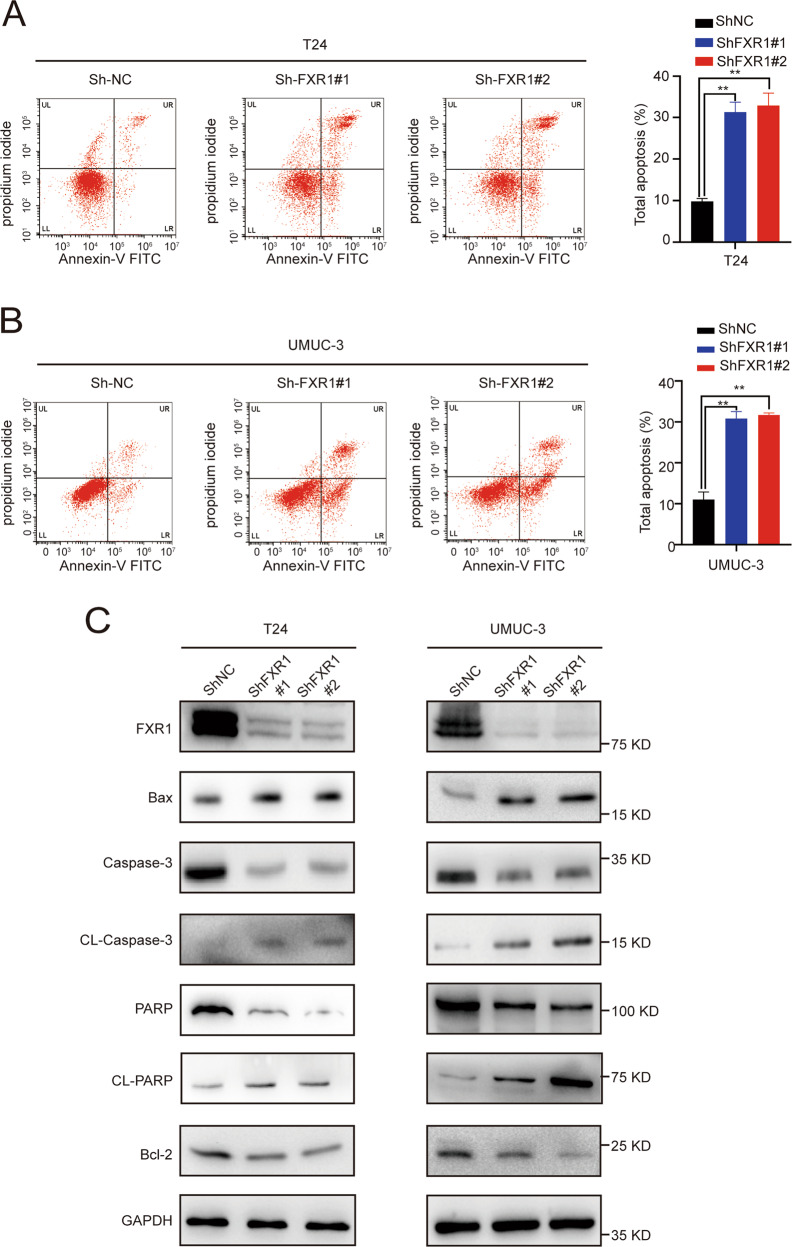
FXR1 inhibits the apoptosis of UCB cells. **A**, **B** Flow cytometry image of control or FXR1 knockdown UCB cells as indicated. Data are presented as the mean ± SD, *n* = 3, **p < 0.01 (Student’s *t*-test). **C** Western blot showing the expression of apoptosis-related proteins in control or FXR1 knockdown UCB cells.

Evasion of cell death is one of the basic changes in a normal cell transformed into malignant cells, and this transformation drives tumorigenesis. Therefore, reduced apoptosis or resistance to apoptosis plays a critical role in carcinogenesis [15]. To verify whether FXR1 is involved in regulating UCB cell apoptosis, annexin V-FITC staining was performed. The percentages of early apoptotic (annexin V-FITC^+^/PI^−^) and late apoptotic cells (annexin V-FITC^+^/PI^+^) were significantly higher in the FXR1 knockdown group than in the control group. The results of the apoptosis detection assay indicated that depletion of FXR1 increased the percentages of early and late apoptotic UCB cells (Fig. 3A, B). Furthermore, the knockdown of FXR1 altered signaling pathways associated with cell apoptosis. The levels of proapoptotic proteins, such as cleaved-PARP, cleaved-Caspase3, and Bax were upregulated, whereas the levels of the antiapoptotic protein Bcl-2 were downregulated in FXR1 depleted cells (Fig. 3C). Hence, these data suggest that FXR1 may promote the escape of UCB cells from the normal cell apoptosis process.

### FXR1 interacts with the TRAF1 mRNA 3′ untranslated region (UTR) and enhances TRAF1 expression

To further explore the molecular mechanisms of FXR1 in promoting UCB oncogenesis, we applied deep RNA-seq and RNA immunoprecipitation sequencing (RIP-seq) to control and FXR1 knockdown cells to identify FXR1 specific targets.

**Fig. 4:**
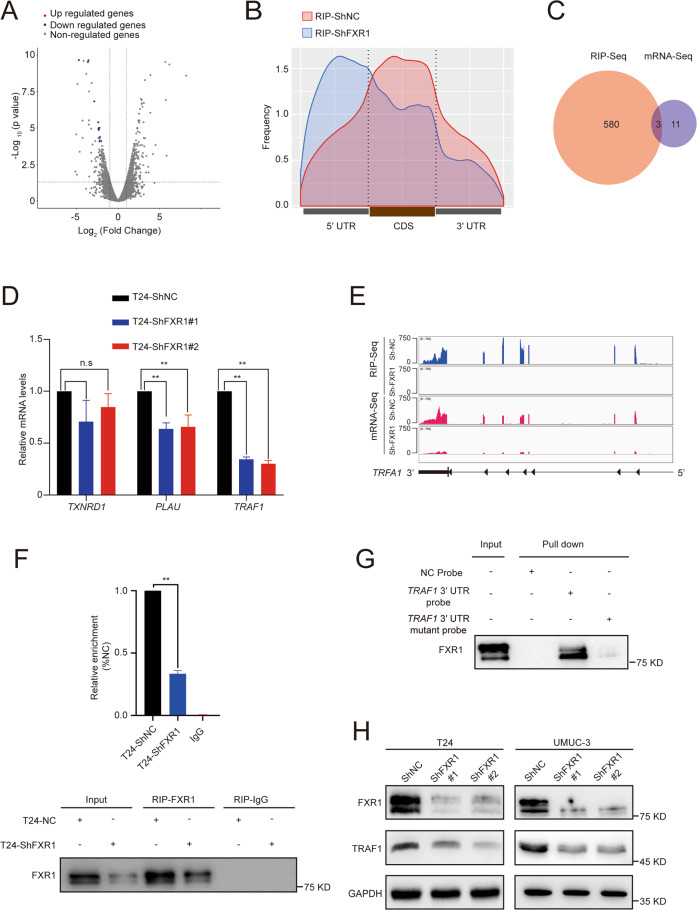
FXR1 affects TRAF1 expression by regulating the 3′ UTR of TRAF1 mRNA. **A** Volcano plot showing the Log_2_ fold change value in UCB cells transfected with control or FXR1 siRNA. Positive values on the *x*-axis indicate the Log_2_ fold change of FXR1 siRNA versus control. The statistical significance (−log_10_ *P*) is shown on the y-axis. Red dots and blue dots represent genes with significant upregulation or downregulation after FXR1 depletion, respectively. **B** Metagene profiles of FXR1 binding sites in control or FXR1 knockdown cells from RIP-seq. **C** The intersection of FXR1-binding target mRNAs and the decreased mRNA after FXR1 knockdown in T24 cells. **D** Extracts of control or FXR1 knockdown T24 cells were analyzed by RT-qPCR using PCR primers specifically amplifying candidate transcripts. Data are presented as the mean ± SD, *n* = 3, **p < 0.01 (Student’s *t*-test). **E** Integrative Genomics Viewer tracks displaying the FXR1 binding site on *TRAF1* transcripts in control or FXR1-depleted T24 cells. **F** RIP and RT-qPCR analysis showed the enrichment of FXR1 on *TRAF1* mRNA (upper panel). Western blot analysis showed the RIP efficiency of the FXR1 antibody (bottom panel). Data are presented as the mean ± SD. **p < 0.01 (Student’s *t*-test). **G** RNA pull-down assay showed the interaction between FXR1 and *TRAF1* 3′ UTR. **H** Western blot analysis revealed TRAF1 protein expression in control or FXR1 knockdown cells.

Through analyzing the transcriptome-wide RNA-seq results, we found that 14 mRNAs were differentially expressed following FXR1 silencing (NC reads > 20, |log_2_ FC | > 2, *p* < 0.05). Most of these genes were downregulated (Fig. 4A). To further identify the transcripts directly regulated by FXR1, we performed RIP-seq experiments using an endogenous anti-FXR1 antibody to enrich the transcripts bound by FXR1. The results showed that FXR1 preferentially binds to the 3′ UTR and coding sequence (CDS) region in the transcripts (Fig. 4B). Of the 583 mRNAs bound by FXR1 in the control cells, these associations with FXR1 were significantly reduced in FXR1-knockdown cells (NC reads > 200, |log_2_FC | > 2, *p* < 0.05). Among these mRNAs, 50.1% contained FXR1 binding sites within the 3′ UTR, and 36% contained FXR1 binding sites mapped to the CDS region. By analyzing the RNA-seq and RIP-seq results, 3 candidates (*TRAF1*, *PLAU*, and *TXNRD1*) were identified (Fig. 4C). Through further qRT-PCR validation, we confirmed that after FXR1 knockdown, *TRAF1* mRNA displayed a remarkable reduction (Fig. 4D). Notably, *TRAF1* was identified to contain an FXR1 binding site in its 3′ UTR (Fig. 4E), suggesting that FXR1 might bind to *TRAF1* transcripts leading to the altered expression of these transcripts. Therefore, *TRAF1* was selected for further investigation.

The interaction between FXR1 and *TRAF1* was confirmed by RIP qRT-PCR and RNA pull-down assays. *TRAF1* mRNA was enriched in FXR1 immunoprecipitated RNAs (Fig. 4F). Next, we constructed a biotin-labeled *TRAF1*-3′ UTR probe and a *TRAF1*-3′ UTR mutant probe whose UGUA motif was changed to ACAG at 2464 after the termination codon. The results showed that FXR1 was pulled down with a biotin-labeled *TRAF1*-3′ UTR probe but not the control probe or the *TRAF1*-3′ UTR mutant probe (Fig. 4G), strongly supporting the association between FXR1 and *TRAF1*-3′ UTR. Subsequently, we measured the protein expression of TRAF1 by transfection of two different shRNAs to knock down FXR1 expression in T24 and UMUC-3 cells. These results resulted in remarkably reduced TRAF1 expression at the protein level (Fig. 4H). In contrast, the expression of FXR1 was not influenced by TRAF1 depletion (Supplementary Fig 3A). With the above evidence, we deduced that FXR1 is the direct upstream regulator of TRAF1.

### FXR1 interacts with cleavage factor I_m_ 25 (CFIm25) and cleavage factor I_m_ 68 (CFIm68) forming a novel complex for mRNA 3′ end processing that functions in sequence-specific poly(A) site recognition

**Fig. 5:**
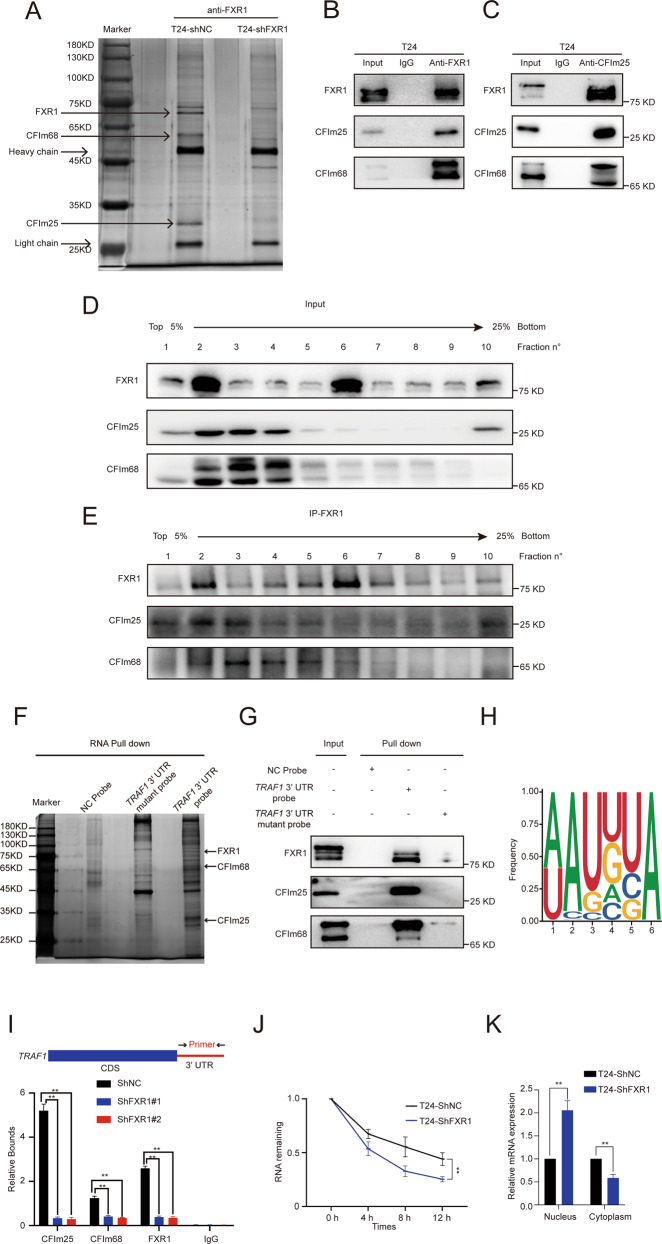
FXR1 recruits CFIm25 and CFIm68 forming a novel complex of mRNA 3′ end processing machinery at the *TRAF1* transcript. **A** Silver staining illustrated the Flag-tagged FXR1 complex in T24 cells. **B**, **C** Co-IP of FXR1-interacting complex or CFIm25-interacting complex in T24 cells. **D** Western blot showing the distribution of FXR1, CFIm25, and CFIm68 sedimented through a 5-25% glycerol gradient in T24 cells. **E** Western blot showing the distribution of the FXR1-interacting complex-sedimented through a 5-25% glycerol gradient in T24 cells. **F**, **G** Silver staining (**F**) and immunoblot (**G**) illustrated *TRAF1* 3′ UTR pull-down protein in T24 cells. **H** UGUA RNA binding motif analysis from FXR1 RIP-seq. **I** ChIP and RT-qPCR analysis showed the recruitment of FXR1, CFIm25, and CFIm68 to the *TRAF1* 3′ UTR in control or FXR1 depleted cells. Data are presented as the mean ± SD, *n* = 3, **p < 0.01 (Student’s *t*-test). **J** qRT-PCR analysis revealed the short half-life of *TRAF1* mRNA in control or FXR1 knockdown cells after treatment with actinomycin D. Data are presented as the mean ± SD, *n* = 3, **p < 0.01 (Student’s *t*-test). **K** qRT-PCR analysis showed the cellular distribution of *TRAF1* mRNA in control or FXR1 knockdown cells. Data are presented as the mean ± SD, *n* = 3, **p < 0.01 (Student’s *t*-test).

To explore how FXR1 regulates *TRAF1* mRNA expression, we screened for FXR1 interacting partners in UCB cells. FXR1 and its associated partners were purified from T24 cells using an FXR1 antibody. Mass spectrometry (MS) analysis identified CFIm25, a core component in mRNA 3′ end processing, as a putative FXR1 partner (Fig. 5A and Supplementary Table S2). It has been reported that the heterodimer of CFIm25 and CFIm68 regulates 3′ end processing [16], so we wondered whether FXR1, CFIm25, and CFIm68 form a complex in UCB cells. A co-immunoprecipitation (co-IP) assay confirmed the interaction among FXR1, CFIm25, and CFIm68 (Fig. 5B). On the other hand, we purified the CFIm25 complex from T24 cells and showed that, beyond the well-characterized core 3′ end processing components (CFIm25 and CFIm68), CFIm25 also interacted with FXR1 in T24 cells (Fig. 5C).

To clarify whether FXR1, CFIm25, and CFIm68 form one or multiple complexes in UCB cells, we fractionated the T24 cell lysates by glycerol gradient sedimentation and performed IP across each fraction. CFIm25 and CFIm68 showed a near-identical distribution ranging from fraction 1 to fraction 9 and peaked between fraction 2 and fraction 4. The results showed a signal peak of FXR1, CFIm25, and CFIm68 in fraction 2 (Fig. 5D). Subsequent IP of FXR1 revealed that both CFIm25 and CFIm68 immunoprecipitated predominantly in fraction 2, indicating that the three proteins form a multiprotein complex in T24 cells (Fig. 5E). The IP of anti-FXR1 from all fractions suggested that FXR1, CFIm25, and CFIm68 co-immunoprecipitated in fractions 2–3. The above research results supported the hypothesis that FXR1, CFIm25, and CFIm68 form a multi-complex in UCB cells. We next investigated whether FXR1, CFIm25, and CFIm68 may function as a 3′ end processing complex on *TRAF1* mRNA. RNA pull-down assays revealed that FXR1, CFIm25, and CFIm68 were pulled down with biotin-labeled *TRAF1*-3′ UTR probes but not the control probe and *TRAF1*-3′ UTR mutant probes, strongly supporting the association among the FXR1/CFIm25/CFIm68 complex and *TRAF1*-3′ UTR (Fig. 5F, G).

CFIm25 and CFIm68 form a core component of the 3′ processing complex and specifically bind to the UGUA element upstream of the polyA signal [17]. Through FXR1 RIP-seq analysis, we found a significant enrichment of FXR1 at the UGUA motif upstream of the polyA signal in the control group, while enrichment was not found in FXR1 knockdown cells (Fig. 5H). Together, these data suggest that FXR1 interacts with CFIm25 and CFIm68, forming a novel 3′ processing component that recognizes the UGUA element upstream of the polyA signal.

### FXR1 depletion in vivo results in the decreased association of CFIm25 and CFIm68 with the TRAF1 locus

Pre-mRNA 3′ processing often occurs co-transcriptionally and is associated with actively transcribed chromatin, and it is critical for new RNA production [18–23]. To investigate whether the FXR1/CFIm25/CFIm68 complex is associated with the actively-transcribed *TRAF1* gene for 3′ processing, we performed chromatin immunoprecipitation (ChIP) with antibodies against FXR1, CFIm25, and CFIm68 with extracts from T24 cells treated with control shRNA or FXR1 shRNA. The resulting immunoprecipitants were tested for enrichment of *TRAF1* locus DNA by quantitative real-time PCR. The association of FXR1 with a DNA fragment spanning the 3′ UTR of *TRAF1* was reduced upon FXR1 depletion (Fig. 5I). Importantly, FXR1 depletion decreased the association of CFIm25 and CFIm68 with the 3′ UTR of *TRAF1* but did not alter the expression levels of CFIm25 and CFIm68 (Supplementary Fig 4A). These results strongly suggest that FXR1 regulates *TRAF1* mRNA levels by facilitating the recruitment of the CFIm25 and CFIm68 components to the *TRAF1* locus for 3′ processing.

### FXR1 is necessary for nuclear stabilization of TRAF1 RNA and affects mRNA 3′ processing

Proper 3′ processing is required for mRNA stability and export. Improperly processed RNA lacking a 3′ polyadenylated tail may become unstable and lead to nuclear retention. Thus, we initially examined whether FXR1 affects *TRAF1* mRNA stability. Notably, after treatment with actinomycin D, the half-life of *TRAF1* 3′ polyadenylated RNA was greatly reduced by FXR1 knockdown (Fig. 5J). We then wondered whether *TRAF1* transcripts were retained in the nucleus upon FXR1 depletion. We isolated nuclear and cytoplasmic RNA fractions and quantified the level of *TRAF1* in each fraction by qRT-PCR. *GAPDH* and *U6* RNA were used as internal references for cytoplasmic RNA and nuclear RNA, respectively. Interestingly, while the distribution of *GAPDH* and *U6* RNA was unaltered by FXR1 knockdown, *TRAF1* mRNA was retained in the nucleus (Fig. 5K). These results together suggest that upon depletion of FXR1, improperly processed *TRAF1* mRNA becomes unstable and is retained in the nucleus.

### FXR1 promotes the proliferation and inhibits the apoptosis of UCB cells via the TRAF1 pathway

**Fig. 6:**
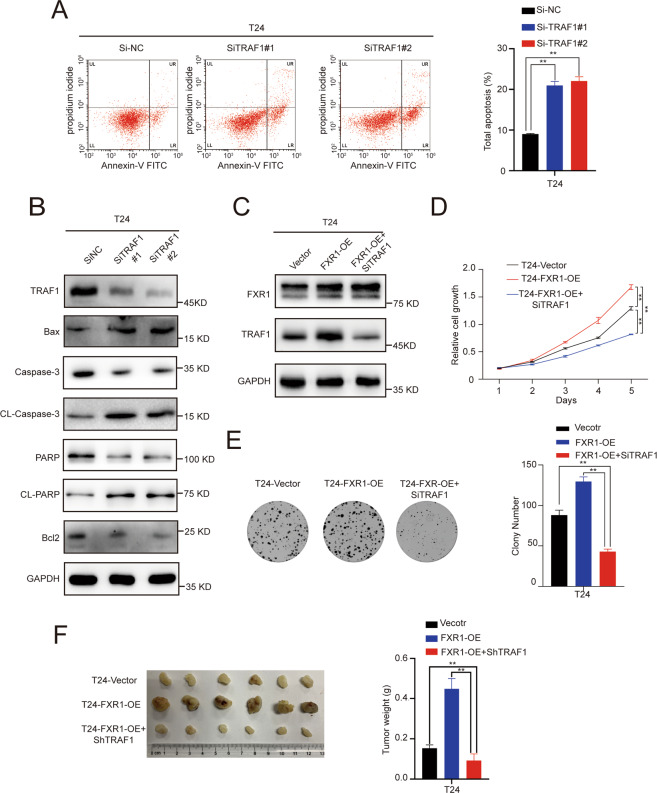
FXR1 promotes UCB pathogenesis by regulating TRAF1. **A** Flow cytometry image of control or TRAF1 knockdown UCB cells as indicated. Data are presented as the mean ± SD, *n* = 3, **p < 0.01 (Student’s *t*-test). **B** Western blot showing the expression of apoptosis-related proteins in control or TRAF1 knockdown UCB cells. **C** Western blot showing the knockdown efficiency of TRAF1 in T24 cells. **D** Growth rate of UCB cells after rescue with TRAF1 in control or FXR1 overexpressing cells. Data are presented as the mean ± SD, *n* = 3, **p < 0.01 (Student’s *t*-test). **E** Represen*t*ative images of colony foci formation in a monolayer culture through rescuing with TRAF1 in control or FXR1 overexpressing cells. The results for quantitative analysis of foci numbers are shown in the adjacent graphs. Data are presented as the mean ± SD, *n* = 3, **p < 0.01 (Student’s *t*-test). **F** Image of the xenograft tumors formed in Balb/c nude mice injected with T24 cells infected with the indicated lentivirus. The weights of xenograft tumors are shown in the right panel. Data are shown as the mean ± SD, *n* = 6 (**p < 0.01, independent Student’s *t*-test).

Next, we investigated whether the FXR1 mediated promotion of UCB oncogenesis is dependent on the TRAF1 pathway. In vitro, we found that knockdown of TRAF1 could mimic the effect of FXR1 depletion, decrease the cell proliferation capacity and increase the percentages of early and late apoptotic UCB cells through the Bcl2/Bax/caspase3 signaling pathway (Fig. 6A, B, Supplementary Fig. 5A, B). In addition, the increases in colony formation and cell proliferation induced by overexpression of FXR1 were largely inhibited upon TRAF1 silencing (Fig. 6C–E). In the Balb/c nude mouse model, we further proved that the enhancement of subcutaneous xenograft tumor growth induced overexpression of FXR1 could be restored by TRAF1 (Fig. 6F). Together, these results suggest that the oncogenic function of FXR1 in promoting the proliferation and inhibiting apoptosis of UCB cells relies on the TRAF1 pathway.

### The co-expression of FXR1 and TRAF1 predicts the worst prognosis for UCB patients

**Fig. 7:**
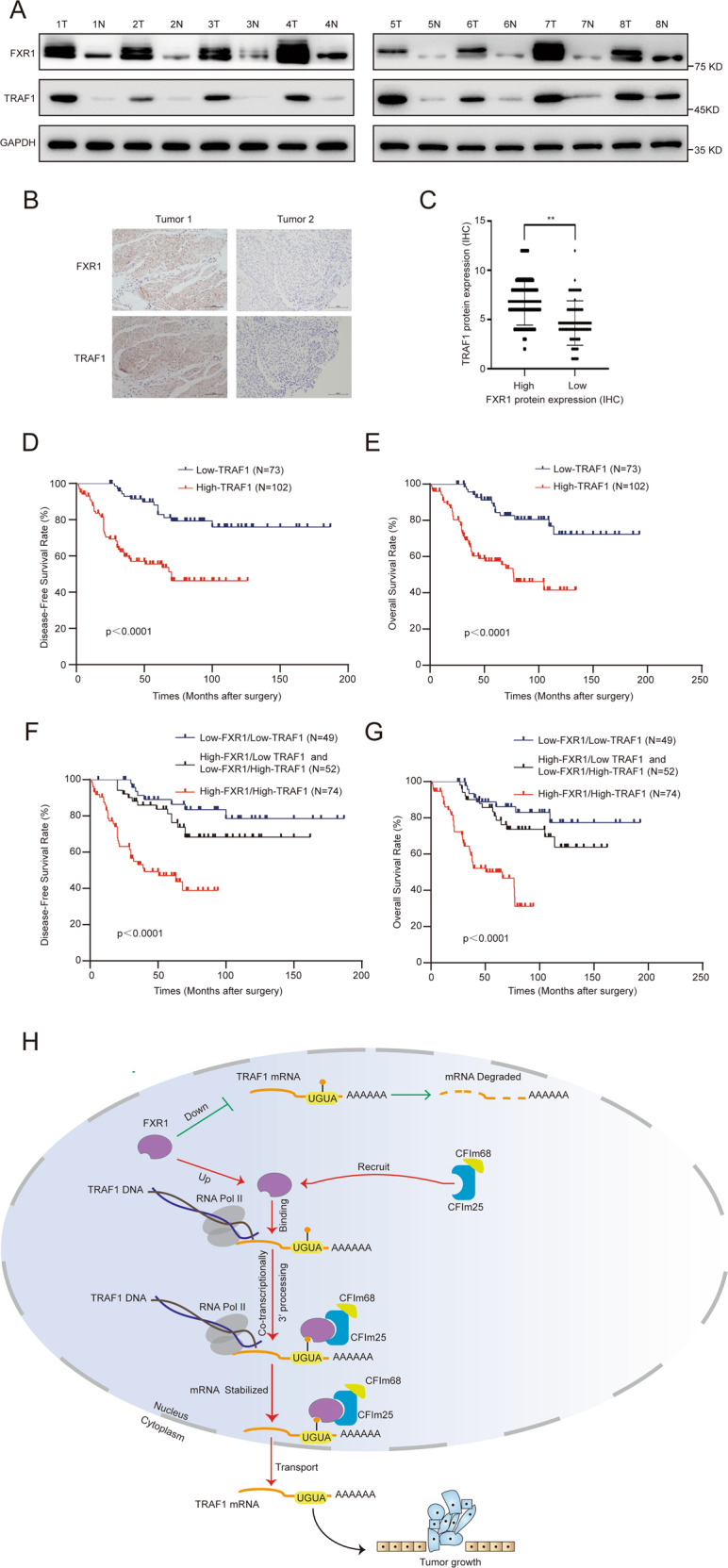
High expression of both FXR1 and TRAF1 predicts the worst prognosis for patients with UCB. **A** Western blot showing the upregulated expression of FXR1 and TRAF1 in eight paired UCB tissues. **B** Representative images of IHC staining showing the correlation of FXR1 and TRAF1 expression in UCB. **C** IHC scores of FXR1 and TRAF1 in tumor tissues. **D**, **E** Survival analysis showing that high expression of TRAF1 predicts poor DFS and OS rates in SYSUCC UCB patients. **F**, **G** Survival analysis shows that high expression of both FXR1 and TRAF1 predicts the worst prognosis in SYSUCC UCB patients. **H** Working model demonstrating that FXR1 regulates the oncogenesis of UCB by regulating the 3′ processing of *TRAF1*.

In order to investigate FXR1 and TRAF1 expression in paired UCB tissues from SYSUCC, we collected another eight paired UCB and paracancerous tissues and found the protein levels of both FXR1 and TRAF1 were frequently upregulated in tumor tissue compared with paracancerous tissue (Fig. 7A).

To investigate the clinical relevance of FXR1 and TRAF1, we performed IHC to detect the protein expression levels of FXR1 and TRAF1 in a cohort of 175 UCB patients from SYSUCC. A positive correlation was found between the expression levels of FXR1 and TRAF1 (Fig. 7B, C). High expression of TRAF1 in UCBs was positively associated with lymph node metastasis, tumor volume, and tumor recurrence (*p* < 0.05; Supplementary Table S3), and a poor prognosis in UCB patients (*p* < 0.05; Fig. D, E). Furthermore, compared to the high expression of either gene alone, high expression of both genes predicted the worst DFS and OS (Fig. 7F, G). These results support the oncogenic role of FXR1 and TRAF1 in UCBs.

## Discussion

RBPs account for approximately 7.5% of protein-coding genes and are known to be involved in all aspects of RNA metabolism [24, 25]. An increasing number of studies have shown that RBPs play an indispensable role in tumorigenesis. Therefore, systematic mapping of the RBP expression landscape in UCB may help us evaluate which RBPs are involved in tumorigenesis and elucidate the underlying pathological mechanisms in UCB. In mammals, RBPs are primarily defined based on their specific RBDs, such as RNA recognition motifs, heterogeneous nuclear RNP K-homology domains, and zinc finger domains. However, the function of most of them is unclear, including whether they actually bind to mRNA or not [26]. Recently, researchers integrated the results of CLIP-Seq and constructed comprehensive protein-mRNA interaction networks containing 104 RBPs, which all contain mRNA binding sites [27]. Here, we built a comprehensive mRNA expression landscape of these 104 RBPs in two independent UCB cohorts from SYSUCC and TCGA. We found more upregulated RBPs than downregulated RBPs in UCB tumors derived from both cohorts. Among 32 consistently upregulated RBPs, *FXR1* and *IGF2BP3* expression displayed a negative correlation with OS in the TCGA UCB cohort (high expression corresponded to poor OS). Importantly, high *FXR1* expression strongly increased the risk of death from disease, suggesting the oncogenic role of FXR1 in UCB.

The upregulation of *FXR1* across most cancer types suggests that *FXR1* exerts conserved or fundamental roles in oncogenesis. FXR1 has been previously shown to be upregulated in head and neck and lung squamous cell carcinoma [28, 29]. However, the underlying mechanism of FXR1 regulation in oncogenesis tends to be complex and varies among various types of cancer. Given that the biological function of FXR1 in UCB remains unknown, it is critical to elucidate the underlying mechanism of FXR1 regulation in UCB. With a series of cell and animal model experiments, we demonstrated that FXR1 strongly enhanced proliferation and inhibited the apoptosis of UCB cells. These findings suggest that FXR1 acts as an oncogenic factor and could be exploited as a drug target for cancer treatment.

To identify cellular targets of FXR1, we applied RNA-seq and RIP-seq for control and FXR1 knockdown cells. From transcriptome-wide RNA-seq of T24 cells, 14 mRNAs were found to be differentially expressed following FXR1 silencing (NC reads > 20, |log_2_FC | >2, *p* < 0.05). Most of these genes were downregulated. By interesting the RNA-seq and RIP-seq results, 3 genes (*TRAF1*, *PLAU*, and *TXNRD1*) were identified. Through qRT-PCR validation, we confirmed that after FXR1 knockdown, the expression of *TRAF1* mRNA, which contains an FXR1 binding site in the 3′ UTR, was significantly reduced.

TRAF1 belongs to the tumor necrosis factor receptor-associated factor family (comprising TRAF1–TRAF7) and is considered to be an adaptor molecule that links upstream receptor signals and gene activation [30]. TRAFs can activate the NF‐κB and JNK, which act as oncogenes, inhibit the apoptosis of cancer cells and promote cancer progression [31–33]. Recent studies reported that high expression of TRAF1 was positively correlated with a poor prognosis in non-small cell lung cancer patients [34]. Depletion of TRAF1 decreased the proliferation capacity and induced the apoptosis of non-small cell lung cancer cells.

One of the most interesting questions was then raised: What is the potential mechanism by which FXR1 regulates TRAF1 expression? The results of this study suggest that FXR1 regulates *TRAF1* transcript processing by binding to CFIm25 and CFIm68 and facilitating their recruitment to transcribed loci of *TRAF1*. IP/MS and glycerol gradient analysis showed that FXR1, CFIm25, and CFIm68 form a triple complex, binding to the UGUA element upstream of the poly(A) site. RNA pull-down and ChIP assays confirmed that the FXR1/CFIm25/CFIm68 complex is associated with the 3′ UTR of *TRAF1* mRNA. We, therefore, concluded that FXR1 regulates *TRAF1* mRNA stability by facilitating the recruitment of CFIm25 and CFIm68 components to *TRAF1* 3′ UTR for 3′ processing.

It remains to be determined whether the FXR1/TRAF1 axis, shown here, is indeed essential to promote tumor formation. We further showed that the effects of enhanced colony formation, cell proliferation, and subcutaneous xenograft tumor growth induced by overexpression of FXR1 were largely inhibited upon TRAF1 silencing in vivo and in vitro. Importantly, in the SYSUCC cohort, UCB patients with high expression of both FXR1 and TRAF1 had the worst prognosis. Together, these results suggest that the oncogenic function of FXR1 in promoting the proliferation and inhibiting the apoptosis of UCB cells relies on the TRAF1 pathway.

In summary, for the first time, we revealed the comprehensive mRNA expression landscape of 104 RBPs in two independent UCB cohorts (SYSUCC and TCGA). We identified *FXR1* as a consistently upregulated RBP in UCB tumors. High FXR1 expression was strongly correlated with reduced OS in the TCGA and SYSUCC UCB cohorts and indicated an increased risk of death from disease. With functional studies, we demonstrated that FXR1 strongly enhanced the proliferation and antiapoptotic capacities of UCB cells. Further mechanistic studies demonstrated that FXR1 regulates *TRAF1* mRNA stability by facilitating the recruitment of the CFIm25 and CFIm68 components to the *TRAF1* 3′ UTR for 3′ processing. Importantly, we demonstrated that the oncogenic function of FXR1 in promoting the proliferation and inhibiting the apoptosis of UCB cells relies on the TRAF1 pathway (Fig. 7H). Our findings further indicate that FXR1 is an effective prognostic biomarker and a new potential therapeutic target for UCB patients.

## Materials and methods

### Patients information

This study involving human tissue was approved by SYSUCC Institutional Review Board and informed written consent was obtained from patients in accordance with the Declaration of Helsinki. All researches involving human participants were blinded.

For IHC, all UCB slide tissues were obtained from 175 patients who underwent radical cystectomy and were pathologically diagnosed with UCB between 2000 and 2013 at SYSUCC. The criteria of the 2016 World Health Organization classification [35] and the eighth edition of the TNM classification [36] of the International Union Against Cancer (2017) were used to define the UCB grade and stage, respectively. All patients were regularly followed up and OS was defined as the time from the date of surgery to the date of death. DFS was elucidated as the time from the date of surgery to the date of the first clinical evidence of cancer recurrence. All samples were formalin-fixed, embedded, and pathologically diagnosed.

For western blot analysis, 16 tumor samples and matched normal tissues were derived from UCB patients who underwent radical cystectomy at SYSUCC between 2015 and 2018.

For organoid culture, UCB tissues were obtained from patients who underwent radical cystectomy at SYSUCC.

### Cell lines and antibodies

The 293T, T24, 5637, J82, UMUC-3, TCC-SUP, and SV-HUC-1 cell lines were purchased from the American Type Culture Collection. The 293T, J82, UMUC-3, TCC-SUP, and SV-HUC-1 cell lines were cultured in RPMI DMEM (Invitrogen, Carlsbad, CA, USA). RPMI 1640 medium (Invitrogen, Carlsbad, CA, USA) was used for T24 and 5637 cell culture. All media were supplemented with 10% fetal bovine serum (HyClone, USA) and 1% antibiotics. All cell lines were tested every 2 months and were not cultured for longer than 2 months. Antibodies used in the study are listed in the Supplementary Methods.

### Animal models

Animal experiments were authorized by the animal ethics committee of SYSUCC. Manipulation of animals followed the approved guidelines in SYSUCC and was conducted with ethical regulations regarding animal research. At least six animals were randomly selected for each condition for analysis. All mice used in our study were purchased from GemPharmatech (Jiangsu, China). To avoid biased manipulation, we used mice of the same sex and similar ages. The xenograft tumor-growth assay was established by the subcutaneous injection of 5 × 10^6^ T24 cells into 4-week-old Balb/c nude mice. Tumor formation in the mice was examined after three weeks.

### Immunohistochemistry

As previously reported [37], IHC was performed with standard streptavidin–biotin–peroxidase complex methods. The final immunoreactivity score (IS) was the product of the staining index and the positive area score (0–4, low expression; 6–12, high expression). The staining index was divided into four levels (negative = 0, weak = 1, moderate = 2, or strong = 3) and the positively stained area score was also divided into four levels (<10%, 1; 10–40%, 2; 40–70%, 3; >70%, 4). The IS of each tissue section was assessed by two independent clinical pathologists.

### RNA-seq

TRIzol reagent (Ambion) was used to extract the total RNA of T24 cells. Dynabeads mRNA purification kit (Ambion) was used for mRNA enrichment. To prepare the transcriptome of each sample, the KAPA mRNA Stranded mRNA-seq kit was used. Subsequently, the transcriptome of each sample was sequenced on the Illumina HiSeq/NextSeq sequencing system (Illumina, California, USA).

### RIP sequencing and qRT-PCR

For RIP, control and FXR1-knockdown T24 cells were harvested and lysed in RIP lysis buffer (Magna RIP RBP Immunoprecipitation Kit, Millipore). After centrifugation, the supernatant was incubated with antibody-conjugated and agarose beads (Roche, USA) overnight at 4 °C. The bead-bound RNAs were extracted with TRIzol and subjected to RIP-seq or qRT-PCR analysis using the primer pairs indicated in Supplementary Table S4. The libraries of immunoprecipitated RNA and input RNA were made using the NEB Next Ultra Directional RNA Library Prep Kit and sequenced on the Illumina HiSeq platform (Illumina, California, USA).

### Bioinformatic analysis

RNA-seq datasets can be accessed through SRA via series PRJNA741055. RIP-seq datasets can be accessed through SRA via series PRJNA741129. All analyses were performed using custom C++ programs.

For RNA-seq analysis, the filtered raw reads were mapped to the GRChg38 genome with FSATQ [38]. The number of reads mapped to each ensemble gene was counted using StringTie [39]. Differentially expressed genes were determined using limma or DESeq2 [40] with a | log_2_FC | cutoff of 1 and false-discovery-rate-value cutoff of 0.05.

For RIP-seq analysis, raw reads were processed using Cutadapt v1.9.1 (length < 35 bp) and Trimmomatic v0.35 [41] to obtain clean reads (length > 35). After alignment with Bowtie2 v2.3.0 [42], Hisat2 v2.0.5 was used to map the clean reads to the GRChg38 genome [43]. Peak detection was conducted by the MACS v2.1.2 [44] peak calling algorithm. And Peak annotation with gene features was executed using BEDTools v2.26.0 [45].

### Organoid culture

Human cancer tissue obtained via surgical resection was stored in serum-free DMEM/F-12 (Invitrogen), which contained 1× Glutamax (Gibco), 20 ng/ml EGF, 20 ng/ml FGF, 10 μΜ Y-27632 (STEMCELL Technologies), 1× gentamicin, 1× amphotericin B, and 1× penicillin–streptomycin. The tissues were cut into 1–2 mm pieces with sterile ophthalmic scissors. After washing with sterile HBSS (Invitrogen), bladder cancer tissue was incubated with collagenase IV (Sigma) for 30 min and then passed through a 100 μm cell strainer (Corning). Cell suspensions were washed twice with DMEM/-F12 complete medium containing 10% serum. Matrigel (Corning) was used to precoat the 24-well plate. After the mixture had solidified, cell droplets were added to 24-well culture plates and incubated at 37 °C.

### Additional methods and original data

Other methods, including plasmid construction and RNA interference, mRNA stability assay, nuclear and cytoplasmic fractionation, glycerol gradient sedimentation, western blotting assays, ChIP, immunoprecipitation, silver stain and MS, RNA pull-down, cell growth, and colony formation assay and flow cytometry and original data, are described in the Supplementary Methods and Supplementary Fig 6.

### Statistical analysis

Statistical analysis for IHC in the SYSUCC cohort was carried out with SPSS 26.0 (IBM Corp, USA) software. The chi-squared test was used to assess the statistical significance of the expression of the indicated protein with the patient’s clinicopathologic parameters and its correlation. Comparisons between groups for statistical significance were performed with the independent sample *t*-test. Survival analysis was performed via Kaplan–Meier curve and Cox hazard analysis. *p* < 0.05 was considered to indicate statistical significance.

In our study, 403 UCB patients from the TCGA cohort were included. Statistical analyses in our study were carried out with R, SPSS 26.0 or GraphPad Prism (version 6.0). The values represent the mean. The error bars represent the SD. The experiments were repeated independently at least three times with similar results.

## Supplementary information

Supplementary Figures

Supplementary Material

Aj-checklist of our study.

## Data Availability

Contact corresponding author for access to materials.
