# Supplementary material for: FXR1 can bind with the CFIm25/CFIm68 complex and promote the progression of urothelial carcinoma of the bladder by stabilizing TRAF1 mRNA
Source: Cell Death Dis. 2022 Feb 22;13(2):170. doi: 10.1038/s41419-022-04614-1 (PMC8863821; doi:10.1038/s41419-022-04614-1)
Supplement: Supplementary file 2 — Supplementary Material [file 41419_2022_4614_MOESM2_ESM.docx]

**Supplementary Material**

| **Supplementary Table S1**: Correlation expression of FXR1 and clinicopathological variables in 175 cases of UCB patients in SYSUCC. | | | | | | |
| --- | --- | --- | --- | --- | --- | --- |
| Variable | All cases  (N=175) | | FXR1 expression (%) | | P value ^a^ | |
|  | | Low expression (%)  (N=77) | | Overexpression (%)  (N=98) | |  |
|  |  |  |  |  |  |  |
| Age(years) |  | |  |  | 0.336 | |
| ≤ 60 | 86 | | 41(47.7) | 45(52.3) |  | |
| >60 | 89 | | 36(40.4) | 53(59.6) |  | |
| Gender |  | |  |  | 0.129 | |
| Female | 21 | | 6(28.6) | 15(71.4) |  | |
| Male | 154 | | 71(46.1) | 83(53.9) |  | |
| Smoking history |  | |  |  | 0.903 | |
| No | 85 | | 37(43.5) | 48(56.5) |  | |
| Yes | 90 | | 40(44.4) | 50(55.6) |  | |
| pT status |  | |  |  | 0.033 | |
| T1 | 33 | | 20(60.6) | 13(39.4) |  | |
| T2-T4 | 142 | | 57(40.1) | 85 (59.9) |  | |
| pN status |  | |  |  | 0.011 | |
| pN- | 142 | | 69(48.6) | 73(51.4) |  | |
| pN+ | 33 | | 8(24.2) | 25(75.8) |  | |
| Histological Grade (WHO, 2004) |  | |  |  | 0.004 | |
| Low grade | 59 | | 35(59.3) | 24(40.7) |  | |
| High grade | 116 | | 42(36.2) | 74(63.8) |  | |
| Tumor volume |  | |  |  |  | |
| ≤ 3 cm | 82 | | 44(53.7) | 38(46.3) | 0.016 | |
| >3 cm | 93 | | 33(35.5) | 60(64.5) |  | |
| Recurrence |  | |  |  | 0.296 | |
| No | 113 | | 53(46.9) | 60(53.1) |  | |
| Yes | 62 | | 24(38.7) | 38(61.3) |  | |
| ^a^ Chi-square test; UCB: Urothelial carcinoma of the bladder. | | | | | | |

| **Supplementary Table S2：List of top 5 candidates of FXR1-interacting protein (25-30KD) that were identified by co-immunoprecipitation and MS.** | | | | |
| --- | --- | --- | --- | --- |
| Gene Name | Unique  Peptides | Coverage  (%) | Molecular  Weight (kDa) | Accession |
| CFIm25 | 14 | 48 | 26,227 | O43809 |
| SRSF1 | 8 | 35 | 27,745 | Q07955 |
| SRSF7 | 6 | 24 | 27,367 | Q16629 |
| SRSF9 | 4 | 21 | 25,542 | Q13242 |
| MYL6 | 3 | 21 | 16,930 | P60660 |

| **Supplementary Table S3**: Correlation expression of TRAF1 and clinicopathological variables in 175 cases of UCB patients in SYSUCC. | | | | | |
| --- | --- | --- | --- | --- | --- |
| Variable | All cases  (N=175) | | TRAF1 expression (%) | | P value ^a^ |
|  | | Low expression (%)  (N=73) | | Overexpression (%)  (N=102) |  |
|  |  |  |  |  |  |
| Age(years) |  | |  |  | 0.868 |
| ≤ 60 | 90 | | 37 (41.1) | 53 (58.9) |  |
| >60 | 85 | | 36 (42.4) | 49 (57.6) |  |
| Gender |  | |  |  | 0.406 |
| Female | 21 | | 7 (33.3) | 14 (66.7) |  |
| Male | 154 | | 66 (42.9) | 88 (57.1) |  |
| Smoking history |  | |  |  | 0.888 |
| No | 85 | | 35 (41.2) | 50 (58.8) |  |
| Yes | 90 | | 38 (42.2) | 52 (57.8) |  |
| pT status |  | |  |  | 0.097 |
| T1 | 33 | | 18 (54.5) | 15 (45.5) |  |
| T2-T4 | 142 | | 55 (38.7) | 87 (61.3) |  |
| pN status |  | |  |  | 0.024 |
| pN- | 142 | | 65 (45.8) | 77 (54.2) |  |
| pN+ | 33 | | 8 (24.2) | 25 (75.8) |  |
| Histological Grade (WHO, 2004) |  | |  |  | 0.652 |
| Low grade | 59 | | 26 (44.1) | 33 (55.9) |  |
| High grade | 116 | | 47 (40.5) | 69 (59.5) |  |
| Tumor volume |  | |  |  | 0.007 |
| ≤ 3 cm | 82 | | 43 (52.4) | 39 (47.6) |  |
| >3 cm | 93 | | 30 (32.3) | 63 (67.7) |  |
| Recurrence |  | |  |  |  |
| No | 113 | | 56 (49.6) | 57 (50.4) | 0.005 |
| Yes | 62 | | 17 (27.4) | 45 (72.6) |  |
| ^a^ Chi-square test; UCB: Urothelial carcinoma of the bladder. | | | | | |

**Supplementary Table S4**: **The primer sequences used to perform quantitative real-time PCR in our study.**

| Primer Name | Direction | Primer sequence (5'-3') |
| --- | --- | --- |
| *TXNRD1* | Forward | ATCAGGAGGGCAGACTTCAAA |
|  | Reverse | TTCACACATGTTCCTCCGAGA |
| *TRAF1* | Forward | CCAGCCTTCTACACTGCCAA |
|  | Reverse | TCACGGTTGTTCTGGTCCAG |
| PLAU | Forward | CTGGGCCTGGGGAAACATAAT |
|  | Reverse | CTGGTTCTCGATGGTGGTGAA |
| TRAF1-3′UTR | Forward | GCTGGGGTGGGTCTCATTAT |
|  | Reverse | GGGAGTTGTTTGTACTGAGCC |
| GAPDH | Forward | TGCACCACCAACTGCTTAGC |
|  | Reverse | GGCATGGACTGTGGTCATGAG |
| U6 | Forward | CAGCACATATACTAAAATTGGAACG |
|  | Reverse | ACGAATTTGCGTGTCATCC |

**Supplementary Table S5**: **The target sequences of si/shRNA used in our study.**

| Gene name | Primer sequence (5′-3′) | |
| --- | --- | --- |
| siTRAF1#1 | 5′-TGTGGAAGATCACCAATGT -3′ | |
| siTRAF1#2 | 5′-GAACCCATCTGTCGCTCTT-3′ | |
| shFXR1#1 | 5′-AGGCCACTAAGCATTTAGA-3′ |  |
| shFXR1#2 | 5′-GCTAGAGGTTTCTTGGAATTT-3′ | |
| shTRAF1 | 5′- GAACCCATCTGTCGCTCTT -3′ | |

**Supplementary methods**

**Plasmid construction and RNA interference**

The pLVX-3*Flag-tagged humanFXR1plasmid was purchased from GeneCreate (Wuhan, China). The short interfering RNAs (siRNAs) and the short hairpin RNAs (shRNAs) were purchased from RIBOBIO (GuangZhou, China) and GeneCopeia (MD, USA), respectively.

Lipofectamine 3000 was used to plasmid and siRNA transfection according to the manufacturer′s instructions (Invitrogen, CA, USA). Lentivirus packaging was performed according to the manufacturer′s instructions (GeneCopoeia, MD, USA). For stable cell construction, lentivirus expressing shRNAs or a gene were infected into cell. Puromycin or neomycin were used for stable cell selection. The relevant interference sequence had been listed in Supplementary Table S5.

**mRNA stability assay**

T24 cells were transfected with siRNAs against FXR1 or control in a six-well plate. 48h after siRNA transfection, the cells were treated with 5 μg/ml Actinomycin D or DMSO, and extracted the total RNA at the indicated time point followed by qRT-PCR analysis. *GAPDH* was used as an internal control.

**Nuclear and cytoplasmic fractionation**

Nuclear and cytoplasmic fractions were extracted as described by the manufacturer, using the reagents supplied in PARIS™ Kit (Thermo Fisher Scientific, Waltham, USA). Briefly, T24 cell was lysed in Cell Fraction Buffer, and centrifuged at 500 × g for 3 min at 4°C. The supernatant was collected as cytoplasmic fraction. The pellet subsequently washed with Cell Fraction Buffer, and centrifuged at 12,000 × g for 10 min at 4°C. The supernatant was collected as nuclear fraction.

**Glycerol gradient sedimentation**

T24 cell extracts were lysed with lysis buffer (HEPES 10 mM, MgCl_2_ 2 mM, KCl 10 mM, NP-40 0.05%, EDTA 0.5 mM, NaCl 150 mM, DTT 1 mM, PMSF 0.1% and 1 × Protease Cocktail). Gradients were centrifuged for 16h at 4℃, 36,000 rpm using a SW41 rotor (Beckham) and fractionated from the top of the centrifugation tubes. Each portion was split into two equal fractions, one part is used as input after extracting protein with methanol and chloroform, the other part is used for immunoprecipitation, respectively. Related proteins are detected by Western blotting.

**Western blotting assay**

Protein extracts were obtained using lysis buffer, and separated on 10% SDS–PAGE, followed by PVDF membrane transfer (Merck-Millipore). Protein extracts were immunoblotted using the indicated primary antibodies and followed by ECL (ThemoFisher).

**Chromatin immunoprecipitation**

For ChIP assays were performed as described previously[1], the control and FXR1-knockdown T24 cells were harvested for cross-link ChIP, which using the antibodies indicated. Samples were amplified by qPCR using the primer pairs indicated. An aliquot of chromatin was amplified in parallel and values obtained for immunoprecipitants were normalized to values for chromatin (% NC). Relative abundance was normalized to the ratio of control cells.

**Immunoprecipitation,** **Silver stain and Mass spectrometry**

For the immunoprecipitation, protein extracts were obtained using RIPA buffer. Protein extracts were incubated with FXR1 antibody conjuncted with agarose beads for 4h at 4℃. The bead-bound proteins were eluted by SDS buffer and subjected to SDS-PAGE. The silver staining was performed followed the instructions of the Fast Silver Stain Kit (Beyotime, Shanghai, China). The protein-containing gel slices were subjected to mass spectrometry analysis (Wininnovate Bio, Shenzheng, China) using Easy nLC 1200 (ThermoFisher, USA).

**RNA pull-down**

The biotin-coupled *TRAF1 mRNA* complex was pulled down with streptavidin-coated magnetic beads (Invitrogen, Carlsbad, USA) in T24 cell lysates following the manufacturer′s instructions. The beads-bound proteins were eluted and analyzed by SDS-PAGE.

**Cell growth and colony formation assay**

For cell growth assay, 1 × 10^3^ cells were cultured in a 96-well plate, and cell growth rate was assessed with the Cell counting kit-8 (MCE, New Jersey, USA).

For colony formation assay, 5 × 10^2^ cells were spread in a 6-well plate and cultured for one week. Cells were fixed with 4% paraformaldehyde for 15 minutes, then stained with crystal violet for 15 minutes and counted.

**Flow cytometry**

For analysis of cell apoptosis, the experiments are carried out according to the instructions of the Apoptosis detection kit (Multi Sciences, Hang Zhou, China). Samples were then processed using the CytoFLEX Flow Cytometer (Beckham, CA, USA). The results were analyzed using CytExpert 2.2 software.

**The antibodies were used in our study**

For western blot: anti-FXR1 (13194-1-AP, 1:1000 dilutions, Proteintech, Chicago, USA), anti-TRAF1 (4173, 1:1000 dilution, Cell Signaling, Boston, USA), anti-CFIm25 (10322-1-AP, 1:1000 dilutions, Protientech, Chicago, USA), anti-CFIm68 (15489-1-AP, 1:1000 dilutions, Protientech, Chicago, USA), anti-BCl2 (4223s, 1:1000 dilution, Cell Signaling, Boston, USA), anti-Bax (2774s, 1:1000 dilution, Cell Signaling, Boston, USA), anti-PARP (9532s, 1:1000 dilution, Cell Signaling, Boston, USA), anti-Cleaved PARP (5625s, 1:1000 dilution, Cell Signaling, Boston, USA), anti-Caspase3 (9662s, 1:1000 dilution, Cell Signaling, Boston, USA), anti-Cleaved Caspase3 (9661s, 1:1000 dilution, Cell Signaling, Boston, USA), anti-GAPDH (6004-1-Ig, 1:5000 dilutions, Proteintech, Chicago, USA).

For IP, RIP and ChIP: anti-FXR1 (13194-1-AP, 1:200 dilutions, Proteintech, Chicago, USA), anti-CFIm25 (10322-1-AP, 1:200 dilutions, Protientech, Chicago, USA), anti-CFIm68 (15489-1-AP, 1:200 dilutions, Protientech, Chicago, USA). anti-IgG for RIP and Chip (2729, 1:200 dilution, Cell Signaling, Boston, USA).

For IHC: anti-FXR1 (13194-1-AP, 1:200 dilutions, Proteintech, Chicago, USA), anti-TRAF1 (4173, 1:200 dilution, Cell Signaling, Boston, USA),

1. Chen, X., et al., *CSTF2-Induced Shortening of the RAC1 3'UTR Promotes the Pathogenesis of Urothelial Carcinoma of the Bladder.* Cancer Res, 2018. **78**(20): p. 5848-5862.
